# Biocompatibility of NeoMTA Plus® versus MTA Angelus as delayed furcation perforation repair materials in a dog model

**DOI:** 10.1186/s12903-021-01552-w

**Published:** 2021-04-13

**Authors:** Khaled M. Abboud, Ashraf M. Abu-Seida, Ehab E. Hassanien, Hossam M. Tawfik

**Affiliations:** 1 Department of Endodontic, Faculty of Dentistry, Ain Shams University, Cairo, Egypt; 2 Department of Surgery, Anesthesiology and Radiology, Faculty of Veterinary Medicine, Cairo University, Giza Square, PO: 12211, Giza, Egypt

**Keywords:** Dogs, Furcal perforation, Inflammatory cell count, Intraradicular bone, Periodontal tissue

## Abstract

**Background:**

The biocompatibility of NeoMTA Plus® (Avlon BioMed Inc., Bradenton, Fl) as a furcal perforation repair material is not fully understood. This study compares the biocompatibility of Mineral Trioxide Aggregate (MTA Angelus) and NeoMTA Plus® as delayed furcation perforation repair materials.

**Methods:**

Pulpotomy and root canal obturation were performed in 72 premolars in six mongrel dogs and then a standardized furcal perforation was performed. The coronal access was left open for three weeks. After curetting, cleaning and drying of the perforations, these teeth were divided into three equal groups (N = 24 teeth/ 2 dogs each) according to the material used for perforation repair; group I: NeoMTA Plus®, group II: MTA Angelus and group III: no material (positive control). The coronal access cavities were sealed with a filling material. The inflammatory cell count and qualitative pathology (presence of calcific bridge, configuration of fibrous tissue formed, examination of tissue surrounding the furcation area, histology of intraradicular bone and the inflammatory nature of tissues) were carried out after one week (subgroup A, N = 8 teeth), one month (subgroup B, N = 8 teeth) and three months (subgroup C, N = 8 teeth). The inflammatory cell count was expressed as mean ± SD and statistically analyzed. *P-*value < 0.05 was considered significant.

**Results:**

In all subgroups, the control group exhibited the highest number of inflammatory cell count, followed by MTA Angelus group and the least inflammatory cell count was shown by NeoMTA Plus® group. There was a significant difference in the inflammatory cell count between the NeoMTA Plus® and MTA Angelus after one week (*P* < 0.05) while no significant differences were recorded between them after one month and three months (*P* > 0.05). In contrast to group II, there was no significant differences in inflammatory cell count between the subgroups in groups I and III (*P* > 0.05). NeoMTA Plus® exhibited better qualitative pathological features than MTA Angelus after one week and nearly similar features after one month and three months of repair.

**Conclusion:**

NeoMTA Plus® has a better early biocompatibility than MTA Angelus after one week of delayed furcation perforation repair and a similar late biocompatibility after one month and three months.

## Background

Root perforation represents a bad complication of endodontic treatment and its treatment is essential to prevent contamination of the surrounding periodontal attachment apparatus and to prevent alveolar bone resorption. The prognosis of perforation repair depends upon several factors such as; presence of bacterial contamination, time and size of perforation and nature of the perforation repair material [1–4].

Furcation perforation has low prognosis because it injuries the periradicular tissues in the furcation area leading to inflammation, granulomatous tissue, bone resorption, periodontal breakdown, epithelial proliferation and periodontal pocket [5]. Furcation perforation could be sealed either by intracoronal nonsurgical treatment or with external surgical access. In both techniques, a good sealing must be carried out between the tooth structure and periodontium [6].

The perforation repair material is of utmost importance, several materials have been applied to be able to meet the biological and mechanical features [2, 3]. Therefore, an ideal perforation repair material is still challenging. The efficacy of a material used for perforation repair depends primarily on its sealability and biocompatibility [4, 7, 8].

The MTA cements are bio-interactive ion releasing alkalinizing materials that have the ability to enhance differentiation of mineralizing cells and nucleation of apatite [9]. The MTA is applied for repair of the root and furcal perforations, root-end filling, endodontic sealing, direct pulp capping and pulpotomies [9]. The MTA has acceptable biocompatibility and enhances the growth of fibroblasts, osteoblasts, cementoblasts, bone marrow stromal cells and pulp cells [10–16]. MTA Angelus is composed of powder (tricalcium silicate, dicalcium silicate, tricalcium aluminate, silicon oxide, potassium oxide, aluminum oxide, sodium oxide, iron oxide, calcium oxide, bismuth oxide, magnesium oxide, insoluble residues of crystalline silica) and liquid (water). The powder is mixed manually with the liquid resulting in calcium hydroxide and calcium silicate hydrate [17, 18].

NeoMTA Plus® is a fine powder new tricalcium silicate material with tantalum oxide (Ta2O5) as a radiopacifying agent instead of bismuth oxide to overcome the discoloration potential [19]. It is mixed with a water-based gel that produces good handling properties. The proportion of mixing powder to liquid can be varied depending upon the indication for use, thin consistency as a sealer or thick consistency as a root end filling or perforation repair material [19, 20]. Furthermore, NeoMTA Plus® has the ability to release calcium, prevent bacterial leakage, adequate radiopacity and a satisfactory sealing ability therefore; it can be used as endodontic sealer or cement repair [8, 21].

The material's properties are fundamental factors for a successful dental treatment. Dentists should always explore different materials to be familiar with newly introduced alternatives and thus provide the best available options to their patients [22]. The effects of NeoMTA Plus® on periodontal ligament cells are not fully understood in terms of biocompatibility. To the authors’ knowledge, the in vivo studies on biocompatibility of NeoMTA plus® are very scarce. The null hypothesis of this study is that NeoMTA Plus® exhibits a similar biocompatibility to the MTA Angelus when used as delayed furcation perforation repair materials. Thus, this study compares the biocompatibility of NeoMTA Plus® and MTA Angelus in a dog model.

## Methods

### Ethical approval

This study was reviewed and approved by the Institutional Animal Care and Use Committee at Faculty of Dentistry, Ain Shams University, Egypt (No: FDASU-Rec-19-11-2017). Also, the authors followed up all guidelines of The Animal Research: Reporting in Vivo Experiments guidelines (ARRIVE).

### Selection of animal model

Six healthy adult mongrel dogs (1–2 years) with intact dentition were selected in this study. The dogs were obtained commercially from Al-Fahad Trading Company for Animals (Abu Rawash, Giza, Egypt). The animals were housed in separate kennels at Faculty of Veterinary Medicine, Cairo University, Egypt and observed two weeks prior to the operative procedures to exclude any diseased dog. They were provided with two meals of cocked food (20 g/kg) and milk and given fresh water ad libitum. The dogs were kept under proper conditions of nutrition, ventilation, clean environment and 12 h light/dark cycle.

### Classification of samples

The second, third and fourth maxillary and mandibular premolars in each dog were utilized for the study (N = 72 teeth). These teeth were divided into three equal groups (N = 24 teeth/ 2 dogs each) according to the material used for perforation repair; group I: NeoMTA Plus®, group II: MTA Angelus and group III: no material (positive control). Each group was further subdivided into three equal subgroups (8 teeth each) according to the post-operative observation period; subgroup A: one week, subgroup B: one month and subgroup C: three months.

### Surgical procedure

Each animal was generally anesthetized by using atropine sulphate at a dose of 0.1 mg/kg given subcutaneously (Atropine®: Sunways Pvt. Ltd., Mumbai, India) then Xylazine (Xylamed®: Bimeda Animal Health, Dublin, Ireland) at a dose of 1 mg/kg given intravenously. General anesthesia was induced by using Ketamine HCl (Ketalar®: JHP pharmaceuticals, Michigan, USA) at a dose of 5 mg/kg given intravenously using a cannula fixed in the cephalic vein and then maintained by Thiopental Sodium (Thiopental sodium®: Livealth Biopharma Pvt., Ltd., Mumbai, India) at a dose of 25 mg/kg 2.5% solution given intravenously.

Standardized periapical radiographs using custom made holding devices were taken to confirm complete root formation and absence of pathologies. After pumice prophylaxis, disinfection of the operative field with 2% chlorhexidine gluconate solution, coronal access cavity was prepared in all experimental and positive control teeth. Exposure of the pulp chamber was obtained through the occlusal surface using #4 round bur with conventional high speed hand piece mounted on a portable air motor. Pulpotomy was performed by a sterile excavator. The working length was determined 2 mm short of the radiographic apex with an electronic apex locator (Root ZXII; J Morita Corp, Kyoto, Japan). Root canal shaping was performed with one shape rotary instruments (MicroMega, Besancon, France) where it was activated by an electric motor (X-Smart; Dentsply Tulsa Dental Specialties) under irrigation with 3.6 mL 1% sodium hypochlorite (NaOCl). After drying the canals with paper points the canals were obturated by lateral condensation of gutta-percha cones and Adseal sealer (EndoSeal, Maruchi, Seoul, Korea).

Perforation was induced in the central region of the pulpal chamber floor with #2 round diamond bur (KG Sorensen, Sao Paulo, SP, Brazil). In all groups, the diameter of the perforation was standardized as being the diameter of the bur used. A new bur was used for every 3 perforations. Hemostasis was achieved with abundant sterile saline irrigation and gentle pressure with sterile cotton pellets. The coronal access was left open for three weeks. The development of an osseous defect surrounding the perforation site was verified clinically and radiographically. For pain control, the dogs were given intra-muscular diclofenac sodium at a dose of 1.1 mg/ kg, once/day for 5 days after surgery [23].

After the infection period, the dogs were re-anesthetized. Under a complete aseptic condition, the perforation site was curetted by a small spoon excavator to remove the debris and inflamed tissues, cleaned with normal saline, and dried with paper points. Treatment of furcal perforations was carried out according to the groups as follows:

The teeth of group I were filled with NeoMTA Plus® (Avalon BioMed. Inc, Bradenton, FL). While those of group II were filled with MTA Angelus (MTA Angelus®, Londrina, Brazil). In groups I and II, the materials were mixed according to the manufacturer’s instructions, carried into the perforation sites by a small amalgam carrier and compacted with a suitable sized plugger. A sterile wet cotton pellet was then placed in the access cavity. Radiographs were taken to confirm the perforation repair. Samples of group III (control) were cleaned by saline irrigation, no repair material was utilized and the defect was sealed by Teflon.

The coronal access cavity of all teeth (experimental and control) was sealed with chemical cured glass ionomer filling material.

The dogs were kept under continuous monitoring for any changes in habits, body weight and food intake during the post treatment evaluation periods.

### Histopathological evaluation

Animals were sacrificed according to the designated observation period by an anesthetic overdose (Thiopental sodium). The maxillae and mandibles were surgically dissected and cut into four quadrants to accelerate the decalcification time. Samples were fixed by formalin, decalcified by placing in formic acid for 14 days then, 17% EDTA solution for 120 days. Each block was trimmed 1 mm away from the edge of perforation in mesiodistal direction in each sample. The filling materials were removed from each tooth. The specimens were washed in running water for 24 h. The specimens were processed by using an open processing system in which the specimens were dehydrated in a series of ethyl alcohol 70%, 95% and absolute alcohol in 18 h. The specimens were then embedded in paraffin wax; sections of each block were cut using a microtome at a setting of 5 micron thickness through the area of the furcal perforation. Slides were stained with hematoxylin and eosin and examined under light microscopy for qualitative and quantitative analysis.

#### Quantitative evaluation (inflammatory cell count)

All images showing the area of furcation defect were captured using digital camera (EOS 650D, Canon, Japan) that was mounted on a light microscope (BX60, Olympus, Japan). Images were then transferred to the computer system for analysis. This was performed in the Precision Measurement Unit, Oral Pathology Department, Faculty of Dentistry, Ain Shams University, Egypt. The histomorphometric analysis was performed using image analysis software (Image J 1.41a, NIH, USA). For inflammatory cell count, 4 fields for each section were taken at an original magnification of × 40.

#### Qualitative evaluation

Stained sections were examined under a microscope at magnification X40 and X4 for detection of presence of calcific bridge, configuration of fibrous tissue formed, examination of tissue surrounding the furcation area, histology of intraradicular bone, and the inflammatory nature of tissues.

### Statistical analysis

The obtained data were collected, tabulated and statistically analyzed using INSTAT statistical analysis software. Numerical data were explored for normality and variance homogeneity using Shapiro–Wilk and Leven’s tests respectively. Data were analyzed using one-way ANOVA followed by Tukey’s post hoc test for intergroup comparisons and repeated measures ANOVA followed by Bonferroni post hoc test for intragroup comparisons. *P-*value < 0.05 was considered significant.

## Results

### Quantitative findings

**Fig. 1:**
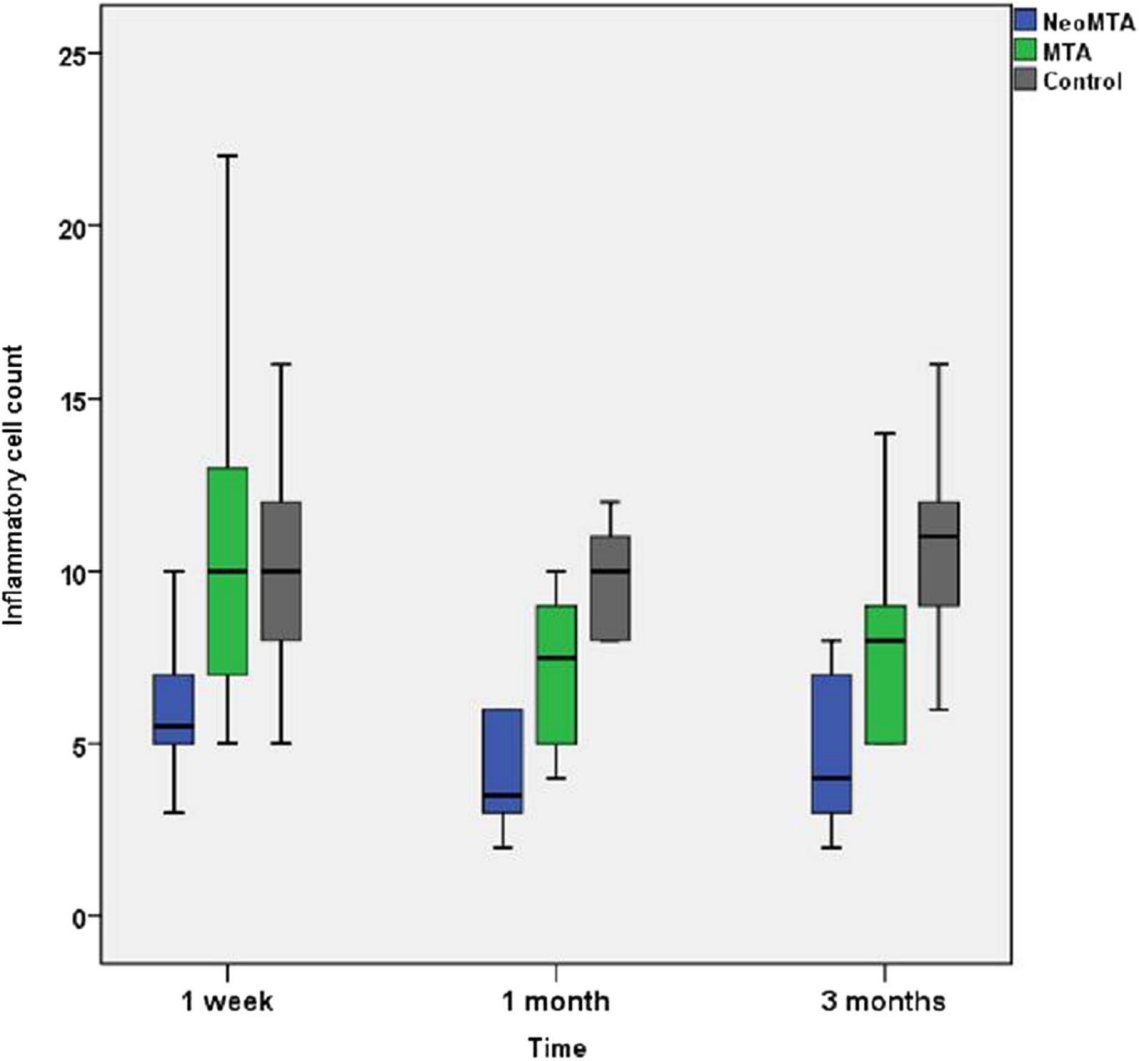
Box plot representation of inflammatory cell count following furcation perforation repair with NeoMTA Plus®, MTA Angelus and control group at different evaluation times

There were significant differences in the inflammatory cell count between the groups (*P* = 0.001) at all evaluation periods. The control group exhibited the highest number of inflammatory cell count, followed by the MTA group and the least inflammatory cell count was shown by the NeoMTA Plus® group as shown in Table 1 and Fig. 1.

**Table 1:** Mean and standard deviation of the inflammatory cell count after perforation repair with the tested materials at different evaluation periods

Subgroups	Groups			
	Group I (NeoMTA Plus®)	Group II (MTA Angelus)	Group III (Positive control)	*P* value
Subgroup A (One week)	6.00 ± 1.94^Ba^	8.60 ± 2.50^Aa^	9.89 ± 2.47^Aa^	0.001*
Subgroup B (One month)	4.87 ± 2.39^Ba^	5.39 ± 2.2^Bb^	9.83 ± 1.72^Aa^	0.001*
Subgroup C (Three months)	5.08 ± 2.02^Ba^	6.92 ± 3.09^Bb^	10.44 ± 2.96^Aa^	0.001*
*P* value	0.289 NS	0.004*	0.861 NS	

Means with different superscript capital letters within the same row are statistically significantly different. Means with different superscript small letters within the same column are statistically significantly different. NS: non-significant; *: significant at *P* ≤ 0.05

In subgroup A (one week), Post hoc pairwise comparisons revealed that the NeoMTA Plus had a significantly lower value than other groups (*P* = 0.001) and the control group had a significantly higher value than other groups in subgroups B (one month) and subgroup C (three months) (*P* = 0.001).

In group I (NeoMTA Plus®), there was no significant difference in the inflammatory cell count at all evaluation periods (*P* = 0.289). The highest amount of inflammatory cell infiltrate was found at one week (6.00 ± 1.94) followed by 3 months (5.08 ± 2.02) while the lowest value was found at one month (4.87 ± 2.39).

In group II (MTA Angelus), there was a significant difference in the inflammatory cell at all evaluation periods (*P* = 0.004). The highest amount of inflammatory cell infiltrate was found at one week (8.60 ± 2.50) followed by 3 months (6.92 ± 3.09) while the lowest value was found at one month (5.39 ± 2.25). Post hoc pairwise comparisons revealed that value measured at one week was significantly higher than that measured at one month (*P* = 0.001).

In subgroup III (positive control), there was no significant difference in the inflammatory cell count at all evaluation periods (*P* = 0.861). The highest amount of inflammatory cell infiltrate was found at 3 months (10.44 ± 2.96) followed by that at one week (9.89 ± 2.74) while the lowest value was found at one month (9.83 ± 1.72) as shown in Table 1.

### Qualitative findings

#### Subgroup A (one week)

**Fig. 2:**
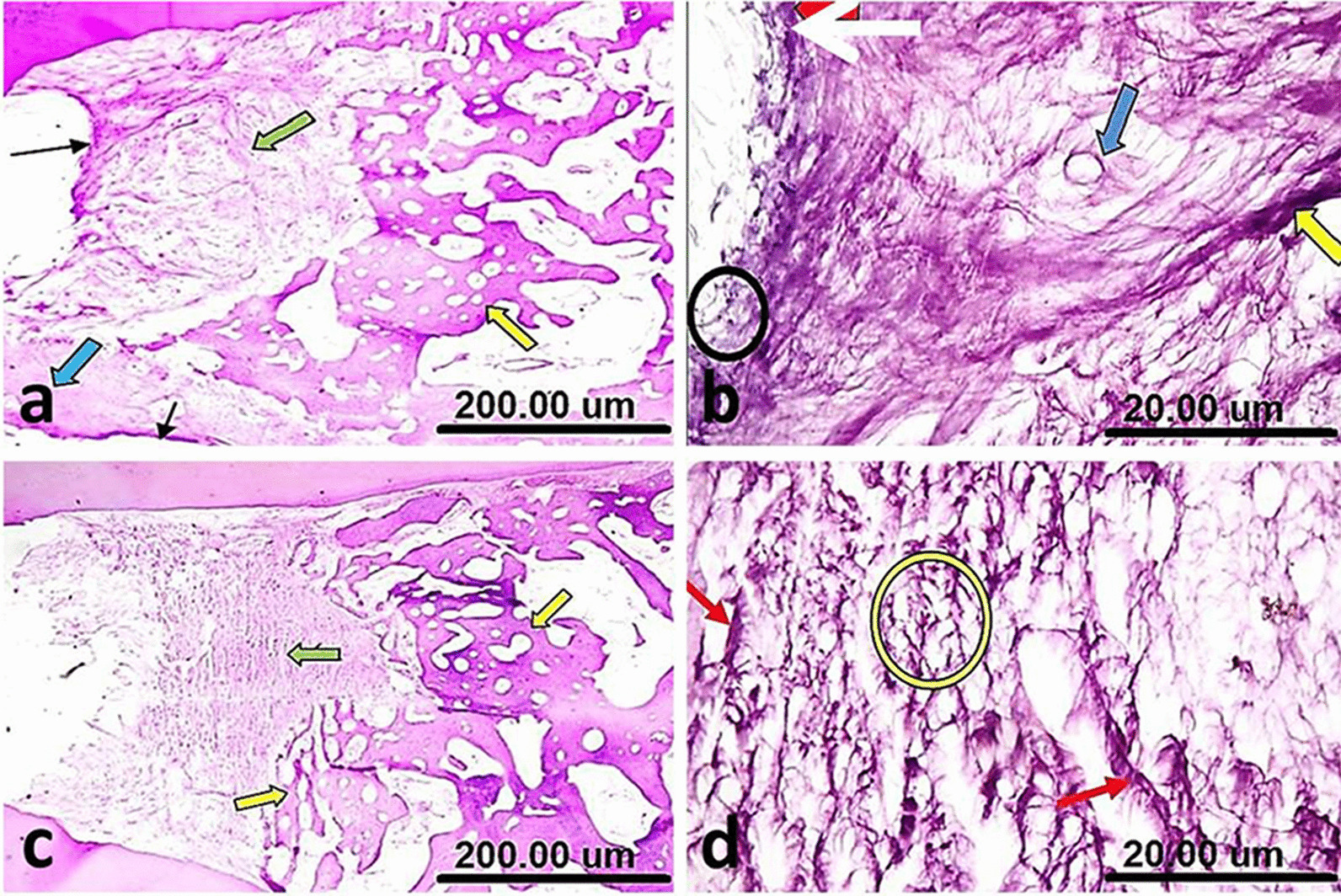
(**a**) A sample of group I (NeoMTA Plus®) after one week showing bridging of the furcation defect by prominent calcific tissue (thin black arrows) that overlying areas of heavily deposited collagen bundles (green arrow), onset of condensation of fibrous tissue into a hyaline matrix of hard tissue formation (blue arrow) and normal histologic morphology (yellow arrow). (**b**) A higher magnification of the same sample in Fig. 2a showing diffuse patchy calcifications at the furcation defect (white arrow), dense collagen fibers (yellow arrow) around blood vessels (blue arrow) and very scanty inflammatory cells (black circle). (**c**) A sample of group II (MTA Angelus) after one week showing plugging of the furcation defect by dense fibrous tissue, horizontally oriented collagen bundles (green arrow) and regular morphology of the interradicular bone (yellow arrows). (**d**) A higher magnification of the same sample in **c** showing fairly calcified fibrous tissue (red arrows), plump fibroblasts and scar tissue formation (yellow circle) [H&E, X4 (**a**, **c**) and X40 (**b**, **d**)]

The samples of group I (NeoMTA plus®) showed bridging of the furcation defect by prominent calcific tissue overlying areas of heavily deposited collagen bundles in different directions. There was a condensation of fibrous tissue into a hyaline matrix of hard tissue formation with very scanty inflammatory cells. The interradicular bone trabeculae had normal histologic morphology, regarding thickness, outline, and cellularity/vascularization (Fig. 2a). Diffuse patchy calcification was also seen at the furcation defect, along the dense collagen fibers and around the blood vessels (Fig. 2b).

In group II (MTA Angelus), there was a plugging of the furcation defect by the formation of a rather dense fibrous tissue with horizontally oriented collagen bundles. Mild evidence for noticeable inflammation was present. The interradicular bone exhibited regular morphology (Fig. 2c). There were fairly calcified fibrous tissue and plump fibroblasts (Fig. 2d).

**Fig. 3:**
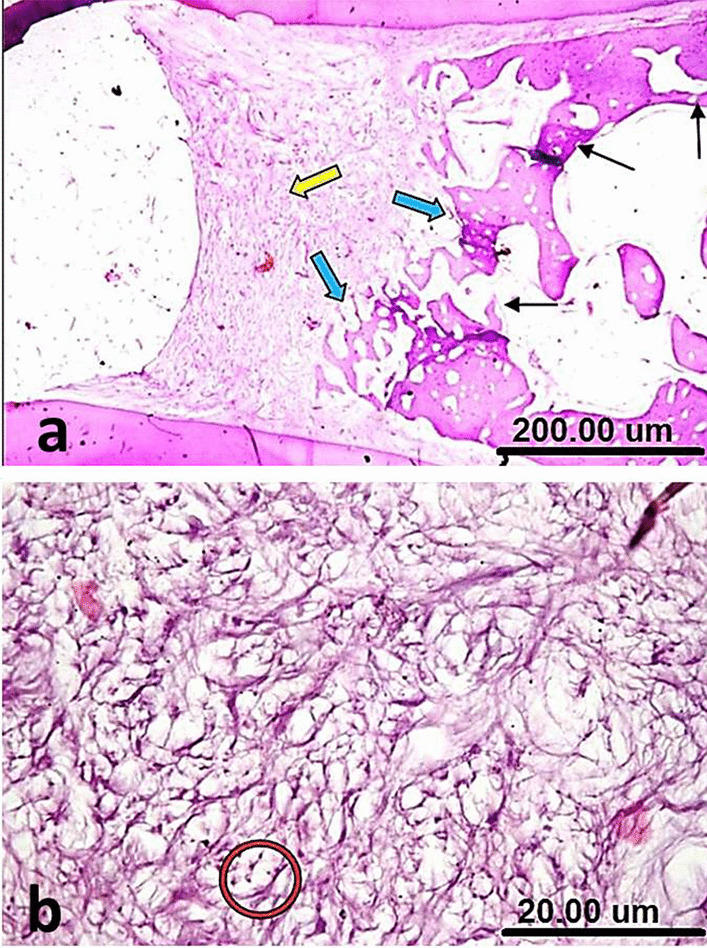
(**a**) A sample of group III (Positive control) after one week showing the furcation defect filled with granulation tissue, no calcific bridge formation (yellow arrow), irregular silhouette of interradicular bone at the granulation tissue interface (blue arrows) and distorted thin discontinuous trabeculae (thin black arrows). (**b**) A higher magnification of the same sample in Fig. 3a showing absence of prominent calcification at the furcation perforation zone and many inflammatory cells scattered among collagen fibers (red circle) [H&E, X4 (**a**) and X40 (**b**)]

In group III (positive control), the furcation defect filled with a granulation tissue with no evident calcific bridge formation. The irregular silhouette of interradicular bone at the granulation tissue interface was also seen besides the distorted thin discontinuous trabeculae (Fig. 3a). Absence of prominent calcification was seen at the furcation perforation zone with many inflammatory cells scattering among the collagen fibers (Fig. 3b).

#### Subgroup B (at one month)

**Fig. 4:**
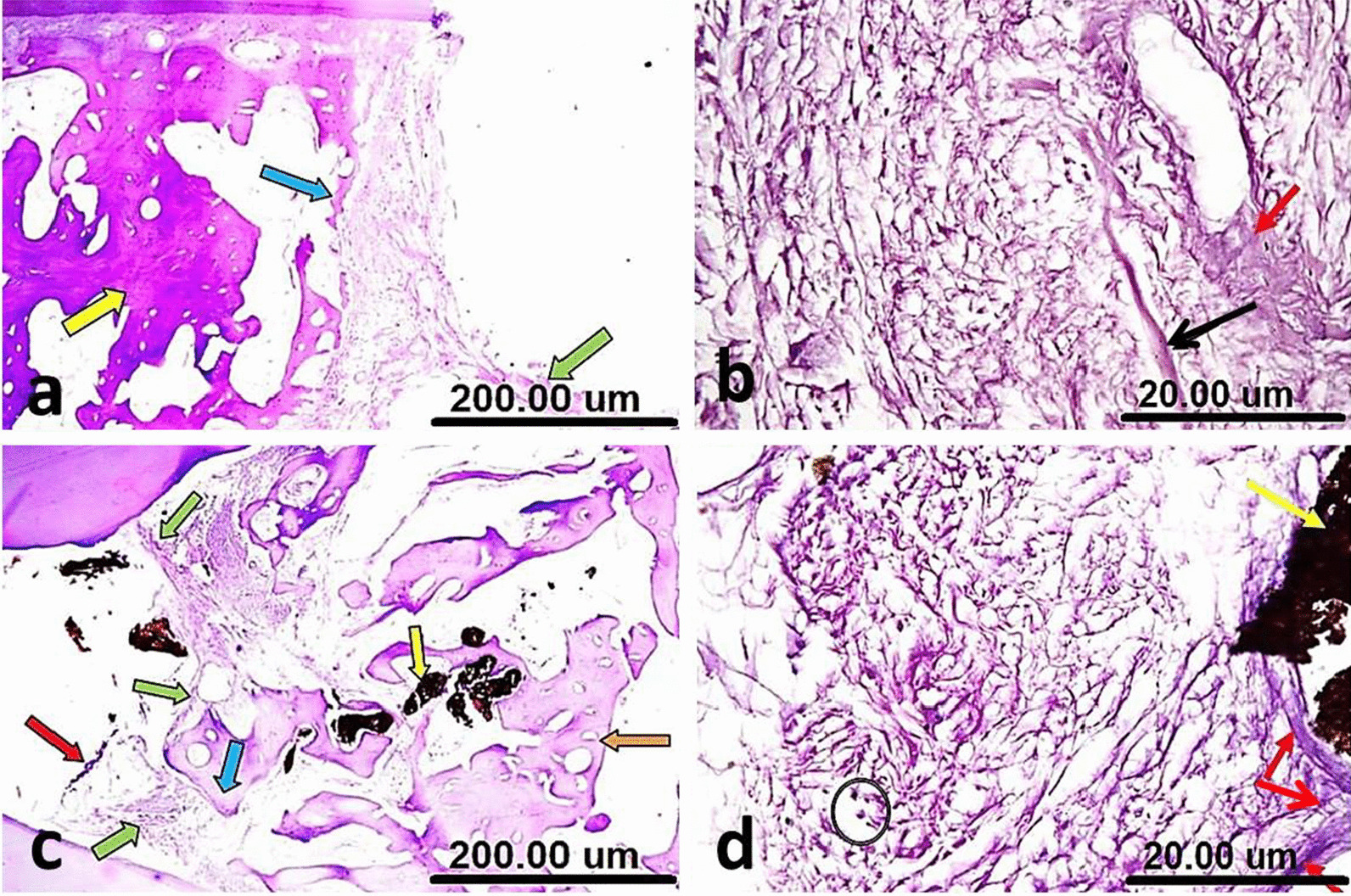
(**a**) A sample of group I (NeoMTA Plus®) after one month showing the area of furcation defect stuffed with coherent fibrous tissue and focal hard tissue formation (green arrow), normal interradicular bone (yellow arrow) and restored uppermost trabeculae (blue arrow). (**b**) A higher magnification of the same sample in Fig. 4a showing a remarkable deposition of calcific tissue around the blood vessel (red arrow) and along some collagen fibers (black arrow) with no inflammatory cells. (**c**) A sample of group II (MTA Angelus) after one month showing condensed fibrous tissue with variable thickness and completeness (green arrows), some lacelike basophilic calcifications (red arrow) and remnants of the repair material (yellow arrow) permeating within the normal trabeculae of interradicular bone (black arrow). (**d**) A higher magnification of the same sample in Fig. 4c showing thread-like calcifications (red arrows) surrounded by the material residues (yellow arrow) and few inflammatory cells (black circle) [H&E, X4 (**a**, **c**) and X40 (**b**, **d**)]

In group I (NeoMTA Plus), the area of furcation defect stuffed with a coherent fibrous tissue, expressing an attempt of bridging via a focal hard tissue formation. The interradicular bone manifested a normal structure and the uppermost trabeculae were restoring their normal orientation and morphology (Fig. 4a). A remarkable deposition of calcific tissue was noticed around the blood vessel and along some collagen fibers with no inflammatory cells (Fig. 4b).

In group II (MTA Angelus), formation of condensed fibrous tissue was seen with variable thickness and completeness. Some lacelike basophilic calcifications were also observed. Remnants of the repair material were noted permeating within the trabeculae of interradicular bone that demonstrated a rather normal configuration (Fig. 4c). Thread-like calcifications were surrounded by the material residues and few inflammatory cells (Fig. 4d).

**Fig. 5:**
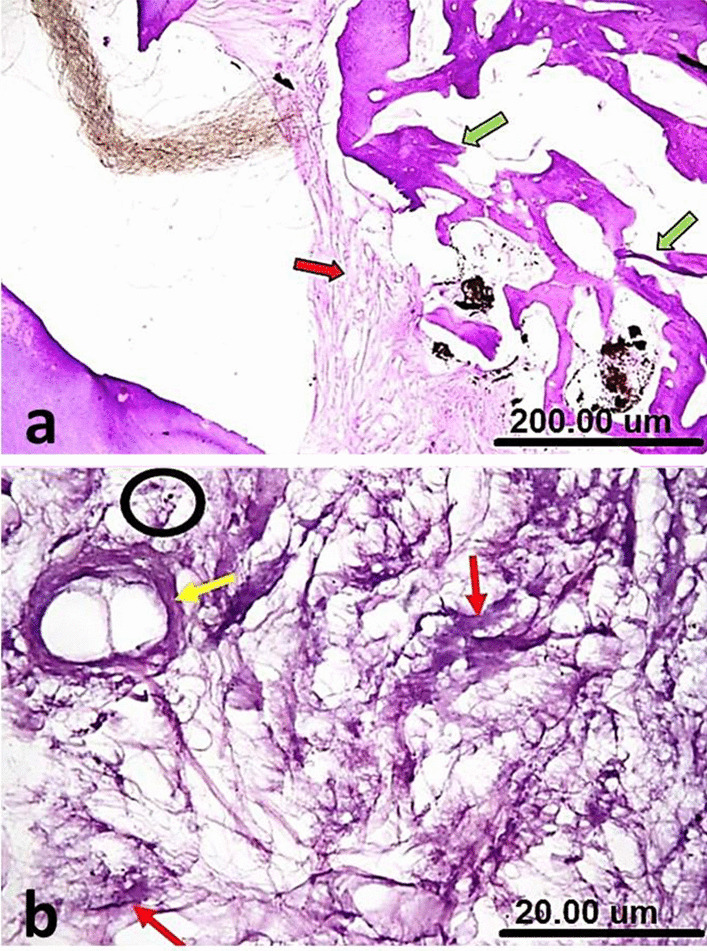
(**a**) A sample of group III (Positive control) after one month showing the furcation defect packed with a poorly compacted fibrous tissue without any trace of hard tissue formation (red arrow) and disfigured interradicular bone with reduced thickness, continuity and cellularity/vascularity (green arrows). (**b**) A higher magnification of the same sample in Fig. 5a showing haphazard calcifications along the randomly oriented collagen fibers (red arrows), calcification around the blood vessel (yellow arrow) and dispersed inflammatory cells (black circle) [H&E, X4 (**a**) and X40 (**b**)]

In group III (positive control), the furcation defect packed with a poorly compacted fibrous tissue without any trace of hard tissue formation. The interradicular bone was clearly disfigured with reduced thickness, continuity and cellularity/vascularity (Fig. 5a). Haphazard calcifications were observed along the randomly oriented collagen fibers. Calcification was noticed around the blood vessel with dispersed inflammatory cells (Fig. 5b).

#### Subgroup C (at three months)

**Fig. 6:**
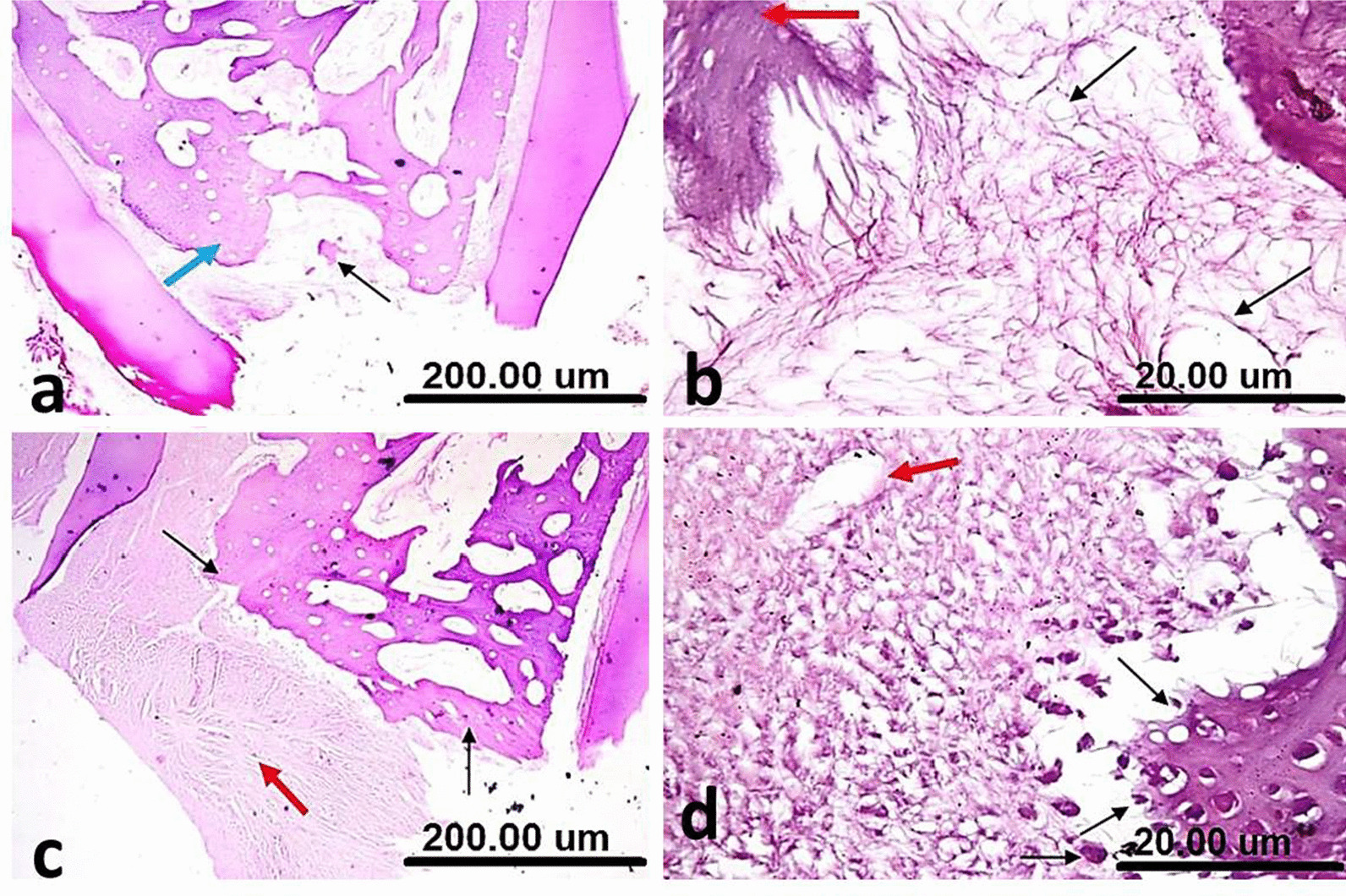
(**a**) A sample of group I (NeoMTA Plus®) after three months showing the furcation defect packed with a fibrous tissue, hard tissue formation (thin black arrow) and almost regained interradicular bone (blue arrow). (**b**) A higher magnification of the same sample in Fig. 6a showing newly formed hard tissue (red arrow), vascular fibrous tissue and no inflammatory cells (black thin arrows). (**c**) A sample of group III (Positive control) after three months showing the furcation defect repaired with a heavy scar tissue, no evidence of hard tissue formation (red arrow) and a preliminary attempt to restore interradicular bone (black thin arrows). (**d**) A higher magnification of the same sample in Fig. 6c showing resorbed interradicular bone with giant osteoclasts, as a part of the remodeling process (thin black arrows) and no calcifications around the blood vessel (red arrow) [H&E, X4 (**a**, **c**) and X40 (**b**, **d**)]

In group I (NeoMTA Plus®), the area of furcation defect packed with a fibrous tissue, exhibiting hard tissue formation. The interradicular bone almost regained its normal shape (Fig. 6a). The newly formed hard tissue was thought to be either a poorly cellular osteoid or osteodentin. The surrounding fibrous tissue was quite vascular and devoid of inflammation (Fig. 6b).

In group II (MTA Angelus)**,** there was hard tissue like structure bridging the perforation defect with hyalinized areas. The hyalinization was seen with mild chronic inflammatory cells around the blood vessels in the underlying connective tissue.

In group III (positive control), the furcation defect repaired with a heavy scar tissue without any evidence of hard tissue formation and the interradicular bone demonstrated a preliminary attempt to restore its regular outline (Fig. 6c). The interradicular bone showed resorption that was mediated by giant osteoclasts, as a part of the remodeling process. No calcifications were seen around the blood vessel (Fig. 6d).

## Discussion

A perforation is a mechanical or pathological communication between the root canal system and the external tooth surface [24]. The use of biocompatible perforations repair material is essential to reduce the incidence of inflammatory reactions in the surrounding tissues [11].

There is a great shortage in the in vivo studies dealing with biocompatibility of NeoMTA Plus® as a furcal perforation repair material. To the authors, knowledge, this study is the first animal study evaluating the biocompatibility of NeoMTA Plus® as a delayed furcation perforation repair material.

The hypothesis of this study is accepted and NeoMTA Plus® can alternate MTA Angelus as a furcal perforation repair material in term of biocompatibility.

In this study, the animal model used was dogs since they have comparative apical repair mechanics with human in shorter duration due to the higher growth rate [1–4]. Dogs have mineral structure and organic responses like those of humans and can withstand long periods of surgical procedure under anesthesia. Therefore, the dog is a commonly used animal model in several previous studies due to these factors [1–4, 13–15].

Any furcal perforation repair material produces favorable outcomes in dogs may have a more favorable results in humans because the dogs, premolars often bifurcate as close as 1–2 mm from the cementoenamel junction (CEJ) [13, 16, 17]. Based on the results of this study, it is expected that the use of MTA Angelus and NeoMTA Plus® as a furcal perforation repair material will produce better results in humans.

The perforation size in this study was standardized at 1.4 mm diameter, which is similar to other previous studies and the bur could penetrate 2 mm into the alveolar bone to enhance the inflammatory response [11, 25, 26]. The other factor that enhanced the formation of the inter-radicular lesion was to leave the perforation site open for saliva contamination for 3 weeks as mentioned before [27].

In the present study, the evaluation periods chosen were one week, one month, and three months. Previous studies have evaluated the healing at intervals shorter than 60 days [28, 29], equal to 60 days [30, 31] and more than 60 days [32]

For evaluation of the furcal perforation healing following repair, the bone loss was assessed in this study through histomorphometric examination and inflammatory cell count using image analysis. Radiography was not used for evaluation of healing in this study because radiographic evaluation was not able to detect tissue response to different treatments after one week while the histologic analysis showed various degrees of osteoplastic and osteoclastic activities that reflect a bony reaction to different treatments [33].

In the current study, the control group showed the highest number of inflammatory cell count at all evaluation periods. This could be attributed to the absence of any repair material. The NeoMTA Plus® group exhibited the lowest inflammatory cell count at all evaluation periods due to its high biocompatibility.

When effect of the time on the inflammation was evaluated, the control group showed no significant differences between the three follow up periods and the highest bone loss and highest inflammatory cell count were recorded after three months. This could be explained by the combined stimulation of bone resorption and inhibition of bone formation by cytokines and prostaglandins [1]. Similar findings were reported by earlier workers [1]. Also, it might be attributed to the chronic inflammation and presence of microorganisms.

In the NeoMTA Plus® and MTA Angelus groups, the inflammation was higher in subgroup A (one week) than subgroups B and C. The predominance of inflammatory infiltrate in the early period could be explained by the release of calcium ions from calcium silicate–based materials. The increase in pH values during setting and heat produced by this reaction enhance inflammatory cell recruitment and production as well as release of proinflammatory cytokines [34–37]. On the other hand, the release of calcium ions and alkalinity of the medium stimulate hydroxyl apatite formation and release of alkaline phosphatase and bone morphogenetic protein 2 that are important in the mineralization process [38]. Moreover, both NeoMTA Plus® and MTA Angelus groups showed high inflammatory cell count initially because the time was not enough to repair the defect. This also agrees with the results of MTA Angelus in previous studies [10, 12, 15, 39].

Both NeoMTA Plus® and MTA Angelus groups showed a significant lower mean inflammatory cell count than the control group due to the sealing ability, biocompatibility and alkaline pH on setting of both materials.

In subgroup A (7 days), the NeoMTA Plus® showed a better biocompatibility than the MTA Angelus and exhibited a comparable biocompatibility after one month and three months of furcation perforation repair. This could be attributed to the adequate radiopacity and prolonged setting time of the NeoMTA Plus®. The ion release and CaP-forming ability could increase stability of the perforation filling and promote endodontic and periodontal tissue regeneration, enhancing the bioactivity and biocompatibility of the material [40].

The results of this study are in agreement with that of Broon et al. [41], who demonstrated complete or incomplete mineralized sealing of the perforations repaired by MTA Angelus in dog´s teeth, with moderate to mild inflammation in the teeth after three months. Also, the results of this study are consistent with the results of Yildirim et al. [14], who found a significant difference in the inflammatory cell count between the MTA Angelus and the positive control group after one month and three months.

A material is considered biocompatible when it promotes cell viability, and the tissue inflammatory response becomes insignificant over time. Accordingly, the NeoMTA Plus® and MTA Angelus showed a suitable biocompatibility after delayed furcation perforation repair in the dog model.

The main limitations of this study were the short follow up periods, lacking of radiographic evaluation of the furcal perforation healing and lacking of histological and cellular components of the newly formed tissues.

## Conclusion

NeoMTA Plus® has a better early biocompatibility than MTA Angelus after one week of delayed furcation perforation repair and a similar late biocompatibility after one month and three months.

## Data Availability

All data used and/or analyzed during this research are available from the corresponding author on reasonable request.

## Abbreviations

MTA: Mineral trioxide aggregate
ARRIVE: The Animal Research: Reporting in Vivo Experiments
Ta_2_O_5_: Tantalum oxide
NaOCl: Sodium hypochlorite
EDTA: Ethylenediaminetetraacetic acid
AAE: American Association of Endodontists
CEJ: Cementoenamel junction
